# Smoking as a predictor of frailty: a systematic review

**DOI:** 10.1186/s12877-015-0134-9

**Published:** 2015-10-22

**Authors:** Gotaro Kojima, Steve Iliffe, Kate Walters

**Affiliations:** Department of Primary Care and Population Health, University College London (Royal Free Campus), Rowland Hill Street, London, NW3 2PF UK

**Keywords:** Frail Elderly, Frailty, Smoking, Tobacco

## Abstract

**Background:**

Evidence on longitudinal associations between smoking and frailty is scarce. The objective of this study was to systematically review the literature on smoking as a predictor of frailty changes among community-dwelling middle-aged and older population.

**Methods:**

A systematic search was performed using three electronic databases: MEDLINE, Embase and Scopus for studies published from 2000 through May 2015. Reference lists of relevant articles, articles shown as related citations in PubMed and articles citing the included studies in Google Scholar were also reviewed. Studies were included if they were prospective observational studies investigating smoking status as a predictor and subsequent changes in frailty, defined by validated criteria among community-dwelling general population aged 50 or older. A standardised data collection tool was used to extract data. Methodological quality was examined using the Newcastle-Ottawa Scale for cohort studies.

**Results:**

A total of 1020 studies were identified and systematically reviewed for their titles, abstracts and full-text to assess their eligibilities. Five studies met inclusion criteria and were included in this review. These studies were critically reviewed and assessed for validity of their findings. Despite different methodologies and frailty criteria used, four of the five studies consistently showed baseline smoking was significantly associated with developing frailty or worsening frailty status at follow-up. Although not significant, the other study showed the same trend in male smokers. It is of note that most of the estimate measures were either unadjusted or only adjusted for a limited number of important covariates.

**Conclusions:**

This systematic review provides the evidence of smoking as a predictor of worsening frailty status in community-dwelling population. Smoking cessation may potentially be beneficial for preventing or reversing frailty.

## Background

Frailty is a multidimensional geriatric syndrome characterised by decreased physiological reserves and increased vulnerability to adverse health outcomes due to age-related accumulation of multisystem deficits and impaired capacity to maintain homeostasis [1–3]. The adverse outcomes include dependency, falls, hospitalisation, institutionalisation and death [2–4]. Although no consensus on a definition of frailty has been reached, the most common description used to operationalise frailty is phenotype criteria proposed by Fried et al. in the Cardiovascular Health Study, the United States [5]. In the study, frailty was defined as having three or more of the following five physical components: unintentional weight loss, self-reported exhaustion, weakness, slow walking speed and low physical activity [5]. Another frequently used approach is the frailty index, a model of accumulated health deficits, which can be constructed based on diseases, symptoms, signs or disabilities [6]. There have been further alternative measurements of frailty proposed in the literature [7], including a range of newer brief measures designed to be used in clinical practice such as FRAIL scale [8] or Edmonton Frail Scale [9].

Smoking is an important modifiable lifestyle factor and has been examined in population-based studies on frailty. However in many studies, smoking has been used for adjustment as a confounding covariate to examine independent risks of target outcomes, and only a limited number of studies have focused on associations between smoking and frailty. Given that tobacco use is a major cause of preventable death and is associated with various negative health outcomes [10, 11], it is hypothesised that smokers are more likely to be frail than non-smokers. Unexpectedly, however, cross-sectional studies show mixed results and in some studies, smoking is associated with being less frail [12–15]. A large European study showed cross-sectional associations between smoking and frailty by age groups [12]. In those in their 50’s current smoking status was positively associated with frailty but negatively associated with frailty for those in their 70’s [15]. In light of higher morbidity and mortality risks in smokers, these paradoxical findings may have resulted from the survivor effect; frail smokers having died early or becoming too frail to smoke, therefore smoking habit as a contributor to frailty may diminish in the very old. In any case, a cross-sectional study design does not allow causal relationships to be inferred and prospective observational studies appropriately controlling for confounding factors are required to assess the causality.

There has been one systematic review paper on the association between frailty and various health-related and socio-demographic factors including smoking [16]. Although ten articles examining smoking and frailty were identified, most of them had a cross-sectional study design and only two articles longitudinally examined smoking as a predictor of frailty changes in the general population [17, 18]. In addition, the review was limited to only studies using Fried phenotype criteria and did not include other important studies using different criteria.

The objective of the current study was to systematically review the literature for evidence on smoking as a predictor of subsequent frailty status changes in longitudinal studies among the general population.

## Methods

### Data sources and search strategy

This systematic review was conducted according to a protocol developed with adherence to Preferred Reporting Items for Systematic Review and Meta-Analyses (PRISMA) statement [19]. One investigator (GK) performed a systematic search of the literature in May 2015 using MEDLINE, Embase and Scopus without language restriction using an explosion function and Medical Subject Heading terms if available from 2000 through current. Validated definitions of frailty were not generally used prior to 2000, and the two most widely accepted definitions and measurements for frailty, the frailty phenotype [5] and the frailty index [6] were first published in 2001. The search terms included (“Smoking” OR “Smoking cessation” OR “Smoking cessation program” OR “Smoking habit” OR “Tobacco” OR “Smokeless Tobacco” OR “Tobacco products” OR “Tobacco consumption” OR “Tobacco dependence” OR “Tobacco smoker” OR “Nicotine” OR “Nicotine derivative” OR “Nicotine gum” OR “Nicotine lozenge” OR “Nicotine Patch” OR “Nicotine replacement therapy” OR “Cotinine” OR “Smok*” OR “Tobacc*” OR “Nicotin*” OR “Cotinin*” OR “Cigarett*”) AND “Frail*”. Additional sources included reference lists of relevant articles, articles shown as related citations in PubMed of the included studies and articles citing the included studies displayed under Cited by in Google Scholar.

### Study selection and data extraction

Studies were considered to be potentially eligible for inclusion if they were prospective observational studies investigating smoking status as a predictor and subsequent frailty status as an outcome among the community-dwelling general population aged 50 or older. In addition, in order to be considered for inclusion, frailty must have been defined criteria originally designed to measure frailty and validated in population-based studies or its modified versions, such as Fried phenotype criteria or the frailty index [5, 6]. Studies were excluded if they substituted other measures, such as disability or nursing home placement [20], to define frailty or used selected samples with certain diseases or conditions [21]. All potentially eligible studies identified were searched for duplicates using the Endnote duplicate finding function and manually, followed by title, abstract and full-text reviews. A standardised data collection tool was used to collect data from the eligible studies.

### Methodological quality assessment

Methodological quality of the eligible studies were examined using the Newcastle-Ottawa Scale for cohort studies [22]. This scale has nine criteria to examine the methodological quality of cohort studies. Each of the included studies was assessed using this scale and considered to have adequate quality if it met five or more of the nine items.

### Data analysis

It was planned to perform meta-analysis to synthesise pooled estimates from the included studies if possible, otherwise a narrative review would be pursued.

## Results

### Selection processes

**Fig. 1:**
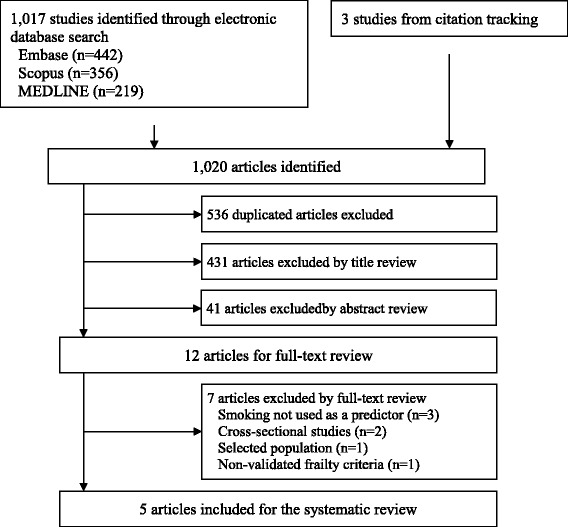
PRISMA Flowchart

A PRISMA flowchart [19] of the literature search and study selection with the number of studies at each stage is presented in Fig. 1. Of the 1020 citations identified from the literature search using three electronic databases and other sources, 536 duplicated studies were excluded, and 431 and 41 studies were also excluded through title and abstract review, respectively, leaving 12 studies for potential inclusion. Full-texts of these 12 studies were assessed and seven studies were further excluded because smoking status was not used as a predictor (*n* = 3), study designs were cross-sectional (*n* = 2), a selected population was used (*n* = 1) or non-validated frailty criteria were used (*n* = 1). Five studies [17, 18, 23–25] were confirmed to meet the inclusion criteria and were included in this systematic review.

### Study characteristics

Characteristics of the five studies are summarised in Table 1. Two studies were from the US [17, 18] and China [23, 24], respectively, and one study used populations from 11 European countries [25]. The largest study involved 28,181 women from the Women’s Health Initiative Observational Study [18]. The other studies used cohorts consisting of almost half men and half women [17, 23–25]. Three studies defined three smoking status categories: ‘never’, ‘past’ and ‘current’ smoking [18, 24, 25] and two studies defined two categories: ‘never/past’ versus ‘current’ smoking [17] and ‘never’ versus ‘past/current smoking’ [23], respectively. Four studies used the Fried phenotype frailty criteria [17, 18, 24, 25]; one study used the frailty index [23]. Although only two kinds of criteria were used, measures of changes in frailty status as outcomes at follow-up varied across the included studies. The follow-up periods ranged widely from two years to 15 years. In terms of statistical analysis, three studies used logistic regression models [18, 24, 25] and two studies used linear regression models [17, 23]. Four studies conducted multivariate regression models controlling for at least age and gender [17, 23–25], which are important confounding factors for both smoking and frailty, and one study showed only the results of unadjusted models [18].

**Table 1:** Summary of included studies on smoking associated with subsequent frailty status change among community-dwelling older people

Author, year	Location	N	Age*	Female (%)	Smoking definition	Frailty outcome	Follow-up	Finding
Woods et al., 2005 [18]	USA	28,181	65–79	100 %	never, past, current smoking	Incident frailty by modified Fried criteria	3 years	- Past smoking was associated with incident frailty (OR = 1.12 95 % CI = 1.02–1.23), but not prefrailty (OR = 0.95 95 % CI = 0.89–1.02).
								- Current smoking was associated with both incidence frailty (OR = 1.76 95 % CI = 1.49–2.09) and prefrailty (OR = 2.90 95 % CI = 2.35–3.57)
								- Unadjusted multinomial logistic regression.
Ottenbacher et al., 2009 [17]	USA	777	82.5	56.4 %	never, past, current smoking	Fried frailty score (range: 0–5)	10 years	- “Ever smoked” was associated with increase in frailty score at follow-up (beta = 0.36, SE = 0.15, *p* < 0.05)
								- Linear regression adjusted for age, gender, education, married, financial strain, diabetes, hip fracture, cancer, stroke, cardiac diseases, arthritis, body mass index and baseline frailty.
Wang et al., 2013 [23]	China	3257	70.1	51.1 %	never, current/past smoking	Frailty index	15 years	- Current/past smoking was associated with increase in frailty at follow-up (beta = 3.64, SE = 1.62, *p* = 0.03) in men.
								- No such association was observed in women.
								- Linear regression adjusted for age, education, baseline frailty index.
Lee et al., 2014 [24]	China	3018	73.6	49.7 %	never, past, current smoking	Change in frailty Category change by Fried criteria	2 years	- No significant association was observed.
								- Gender-stratified age-adjusted logistic regression
Etman et al., 2015 [25]	11 European countries	14,082	>55	54.3 %	never, past, current smoking	Worsening in frailty by Fried criteria (robust > prefrail/frail or prefrail > frail)	2 years	- Current smoking was associated with worsening of frailty status at follow-up (OR = 1.16, 95 % CI = 1.02–1.32, *p* < 0.05)
								- Logistic regression adjusted for age, gender, education, baseline frailty and country.

*Mean age, age range, or age for inclusion

95 % CI: 95 % confidence interval, OR: Odds ratio, SE: Standard error

The included studies were assessed for methodological quality using the Newcastle-Ottawa quality assessment scale for cohort studies. All five studies met at least five criteria and were considered to have adequate methodological quality (Table 2).

**Table 2:** Methodological quality assessment using the Newcastle-Ottawa Quality Assessment Scale for cohort studies

Author	Selection 1	Selection 2	Selection 3	Selection 4	Comparability 1	Comparability 2	Outcome 1	Outcome 2	Outcome 3	Total
Woods et al., 2005 [18]	1	1	0	1	0	0	1	1	0	5/9
Ottenbacher et al., 2009 [17]	1	1	0	n/a	1	1	1	1	0	6/8
Wang et al., 2013 [23]	1	1	0	n/a	1	1	1	1	0	6/8
Lee et al., 2014 [24]	1	1	0	n/a	1	0	1	1	0	5/8
Etman et al., 2015 [25]	1	1	0	n/a	1	1	1	1	0	6/8

Etman et al. investigated associations between smoking status (never, former and current) at baseline and frailty status at two-year follow-up using a large cohort of 14,082 middle-aged and older community-dwelling men and women from the Survey on Health, Ageing, and Retirement in Europe (SHARE) [25]. Using modified Fried phenotype criteria (either from robust to prefrail/frail or from prefrail to frail), the authors showed that current smokers had a 16 % increased risk of worsening frailty status two years after baseline, compared to those who never smoked; multivariate logistic regression models were adjusted for age, gender, educational level, baseline frailty state and country (OR = 1.16, 95 % CI = 1.02–1.32, *p* < 0.05).

In the Hispanic Established Populations for Epidemiologic Studies of the Elderly (EPESE), among 777 Hispanic Americans aged 65 or older, those who ever smoked were significantly more likely to have a higher frailty status at follow-up than those who never smoked [17]. In this study, a summary frailty score, defined as the total number of five components of Fried phenotype criteria ranging from 0 to 5, was created and used as a continuous variable in multivariate linear regression models adjusted for age, gender, body mass index, education, marital status, financial strain, chronic diseases and baseline frailty score to examine frailty status changes over 10 years (unstandardised coefficient = 0.36, standard error = 0.15, *p* < 0.05).

A Chinese study of 3018 community-dwelling older people examining changes in frailty status over two years according to smoking status is the only study that failed to show significant findings [24]. Although not reaching statistical significance, directions of the associations between smoking and frailty appear consistent with the other included studies in that frailty status of (male) current smokers were more likely to worsen and less likely to improve than it was for those who never smoked in age-adjusted logistic regression models (OR = 1.53, 95 % CI = 0.73–3.23 for prefrail worsening; OR = 1.29, 95 % = 0.75–2.23 for robust worsening; OR = 0.63, 95 % = 0.33–1.21 for prefrail improvement; OR = 0.21, 95 % = 0.02–1.80 for frail improvement). No trends were observed among women. There is a possibility that the statistical power may have been lost as a result of dividing the cohort by gender and further by three Fried frailty categories (robust, prefrail and frail) at baseline as well as using three smoking statuses as predictors (never, past and current) and using four different frailty transition states (prefrail worsening, prefrail improvement, robust worsening and frail improvement).

A US study involving 28,181 women aged 65 to 79 from the Women’s Health Initiative Observational Study who were free from frailty at baseline examined risk of newly developing frailty and prefrailty with modified Fried phenotype criteria over three years according to baseline smoking status and using unadjusted multinomial logistic regression models [18]. Past smoking predicted incident frailty (OR = 1.12, 95 % = 1.02–1.23), but not prefrailty (OR = 0.95, 95 % CI = 0.89–1.02), and current smoking predicted incident frailty (OR = 2.90, 95 % CI = 2.35–3.57) and prefrailty (OR = 1.76, 95 % CI = 1.49–2.09). The findings of this study need to be interpreted cautiously because important confounding factors including age, socioeconomic status, education and alcohol use, were not controlled for in the models.

Only one study employed a frailty index and assessed frailty status among 3257 Chinese community-dwellers aged ≥ 55. Men and women were analysed separately using multivariate linear regression models adjusted for age, education and baseline frailty index [23]. Current and past male smokers showed a worsening in their frailty status over the 15-year follow-up, significantly more than men who never smoked (standardised coefficient = 3.643, standard error = 1.621, *p* = 0.026) while there was no such difference observed in women (*p* = 0.529). In this study, the frailty index was constructed based on 28 variables excluding respiratory health deficits such as chronic tracheitis or cough, which are directly related to smoking. The analyses were also repeated with a frailty index using 25 variables without three non-respiratory smoking-related variables (hypertension, cardiovascular disease and cerebrovascular disease), providing similar results.

Most studies demonstrated current, past (or both) smoking status at baseline predicted subsequent incident or worsening of frailty status at follow-up [17, 18, 23, 25]. One study failed to show any significant associations between baseline smoking status and frailty trajectories [24]. It is of note however that most of the estimate measures were either unadjusted or only adjusted for a limited number of important covariates. We were unable to perform a meta-analysis due to methodological diversity of the included studies.

## Discussion

This systematic review identified five prospective cohort studies on smoking and frailty. Although the studies employed different methodology and frailty criteria, most studies demonstrated that baseline smoking significantly predicted worsening of frailty status at follow-up. All studies at best only adjusted for a very limited range of potential confounding factors.

The association between smoking and subsequently developing or worsening frailty demonstrated by the included studies suggests that smoking may play a role in the pathogenesis of frailty. The underlying mechanism by which smokers are predisposed to frailty is not clear but is likely to be multifactorial given the detrimental effects of smoking on a wide range of organs and tissues [11]. Smoking is associated with cardiovascular diseases, respiratory diseases and cancers [11], all of which could cause morbidities and disabilities (both physical and mental), and potentially contribute to increased risks of frailty status.

The association between smoking and frailty may be explained by inflammation. Cigarette smoke contains various toxic chemicals and has been shown to be associated with increased levels of various inflammatory mediators [26]. Chronic inflammation causes muscle wasting [27] and leads to weight loss, exhaustion, weakness or slow gait speed; these are all major components of frailty [5]. This possible link between smoking and frailty via inflammation is further supported by population-based studies reporting that elevated inflammatory markers were associated with a higher prevalence and incidence of frailty [28–30].

The current systematic review has some limitations. First, the systematic literature search, study selection, data extraction and methodological quality assessment were conducted by one researcher; involving at least two researchers would have been more appropriate. Second, a relatively limited number of studies were identified, and some studies may have been missed that were not referenced on the three main data sources searched. Nonetheless, four out of the five included studies consistently showed evidence that smoking was a predictor of frailty status. Third, partly because a uniform definition of frailty has not yet been identified, study designs and methodologies of the included studies varied widely therefore meta-analysis was not possible.

“Currently smokers” can range from a person who smokes a few cigarettes a day to a person who has been smoking two packs per day for five decades, and “former smokers” can be a fit person who temporarily smoked when he/she was a teenager or can be a frail person who had to quit smoking recently because of severe emphysema due to life-long heavy smoking. All of the included studies examined only current status of smoking, however the amount of smoking history, such as by pack-years, is an important factor to examine impacts of smoking, and none of the studies in this review examined this. Therefore, the magnitude of the contribution of smoking to the development of frailty was not clear from the evidence identified by the current review. One study cross-sectionally investigated severity of frailty across three groups created based on amount and length of smoking history: 1) heavy smokers defined as one pack a day for 20 years or more, 2) light smokers defined as less than one pack a day or 1 pack per day for less than 20 years and 3) never smokers, and showed a dose-response association between smoking and frailty: heavy smokers had the highest degree of frailty and never smoker the lowest [15].

Although some of the included studies were not originally designed to examine the associations between smoking and frailty, it is important to note that some studies did not adjust or only adjusted for a limited number of confounding factors [18, 24, 25]. The important variables which should be considered for the link between smoking and frailty may include but not limited to age, gender, education, socioeconomic status and alcohol use.

In the future research, therefore, detailed smoking history information in addition to current smoking status, rather than just current, past and never smoking, and controlling for the abovementioned confounding variables should be taken into account to enable more accurate analysis and to provide more relevant results on the association between smoking and frailty.

## Conclusion

In summary, this systematic review provides evidence suggesting smoking can be a predictor of worsening frailty status among community-dwelling people. Smoking cessation may potentially be beneficial for preventing or reversing frailty.

## Abbreviations

EPESE: Established Populations for Epidemiologic Studies of the Elderly
SHARE: the Survey on Health, Ageing, and Retirement in Europe
